# Obtaining Muramic Acid from *Staphylococcus aureus*: A Simple Strategy for Axenic Isolation of *Tannerella forsythia*

**DOI:** 10.3390/life15121901

**Published:** 2025-12-12

**Authors:** Tanya Pereira-Riveros, Felipe Aguilera, Josep M. Sierra, Damaris Berbel, Teresa Vinuesa

**Affiliations:** 1 Microbiology Unit, Department of Pathology and Experimental Therapeutics, School of Medicine and Health Sciences, Institut Investigacio Biomedica Bellvitge, University of Barcelona, L’Hospitalet de Llobregat, 08907 Barcelona, Spain; tanyapereirariveros@gmail.com (T.P.-R.); faguileramunoz@gmail.com (F.A.); jmsierra@ub.edu (J.M.S.); 2 Department of Microbiology and Parasitology, Hospital Universitari de Bellvitge IDIBELL, University of Barcelona, L’Hospitalet de Llobregat, 08907 Barcelona, Spain; dberbelp@bellvitgehospital.cat

**Keywords:** *Tannerella forsythia*, *Staphylococcus aureus*, periodontopathogen, muramic acid, peptidoglycan

## Abstract

**Background:** The periodontal pathogen *Tannerella forsythia* is auxotrophic for muramic acid (MurNAc), a key component of bacterial peptidoglycan, and dependent on an external supply of MurNAc to maintain pure laboratory cultures. The focus of this study was to find a source of muramic acid and peptidoglycan fragments from a *Staphylococcus aureus* strain. This would facilitate the isolation of *T. forsythia* by incorporating peptidoglycan into conventional anaerobic media. **Methods:** The *S. aureus* strain ATCC 29213 was chosen as the source. The standardization and quantification of the method included verifying concentrations via spectrophotometry and developing a linear regression model with standard curves for muramic acid and lactic acid. The resulting lysate was used to seed Fastidious Anaerobe Agar (FAA) plates, which were inoculated with strain *T. forsythia* (ATCC 43037) and incubated in an anaerobic chamber for seven days. **Results:** The resulting lysate had an optical density ranging from 0.061 to 0.083, which corresponds to a muramic acid concentration of approximately 12 µg/mL. Pure cultures of *T. forsythia* could then be obtained on FAA plates supplemented with muramic acid (MurNAc) (FAA-Mur). The viability of the axenic *T. forsythia* culture was confirmed using muramic acid/peptidoglycan fragments of microbial origin. **Conclusions:** The method presented improves the growth of *T. forsythia*. Consequently, *T. forsythia* is available for further investigation into the regular performance of sensitivity tests in periodontics and the routine generation of growth curves for quantitative polymerase chain reaction (qPCR) analysis.

## 1. Introduction

Periodontitis is a chronic inflammatory disease with multiple causes that leads to the destruction of the tissues that support the teeth [1,2,3,4]. Its pathogenesis is related to an inappropriate immune response of the host, which is closely linked to the presence of a complex microbial community in dental plaque, especially, but not only, the “red complex” described by Socransky, which consists of highly virulent, culturable, gram-negative bacteria: *Porphyromonas gingivalis, Treponema denticola*, and *Tannerella forsythia* [5,6].

In recent years, multiple investigations on *P. gingivalis* have provided a great deal of information regarding its pathogenicity [7,8,9,10]. This is probably due to the fact that isolating *P. gingivalis* under strict anaerobic conditions is much simpler than isolating the other two members of the red complex, *Treponema denticola* and *Tannerella forsythia*. Culturing these two microorganisms is challenging due to their strict nutritional requirements, which makes their laboratory isolation difficult [11,12,13].

*T. forsythia*, first isolated by Tanner [14], has been associated with advanced forms of periodontal disease, such as severe and refractory periodontitis, as well as the transition from periodontal health to disease [15,16,17]. The presence of *T. forsythia* in the oral cavity has been demonstrated to be associated with periodontitis, as well as with endodontic diseases, halitosis, and peri-implantitis. Although *T. forsythia* has been identified in some healthy subjects, this carrier state appears to be merely a preliminary step in the development of the aforementioned pathologies. Furthermore, it has been hypothesised that *T. forsythia* plays a role in the aetiology of periapical abscesses, particularly in cases of symptomatic primary endodontic infections. This is achieved by inducing inflammatory responses in immune cells, resulting in the loss of soft and hard tissue structures that provide support to teeth in the later stages of the disease [18,19,20]. Therefore, its detection should not go unnoticed in any microbiological laboratory supporting clinical practice.

Their involvement in the pathogenesis of periodontitis, particularly aggressive forms of the disease, may be due to their virulence factors [21]. These factors include protease and apoptosis-inducing activities, which facilitate tissue destruction [22]; surface-associated glycoproteins, which promote adhesion and modulate the immune response [23]; and glycosidic activity, which contributes to inflammation [24,25,26].

In addition, *T. forsythia* can shuttle sialic acid into the peptidoglycan synthesis pathway, which is an important metabolite for the bacterium’s biofilm growth [27,28]. The bacterium produces the sialidase enzymes NanH and SiaH1, which release free sialic acid from glycoconjugates. This acid can then be utilized by bacteria [29]. NanH-dependent release of sialic acid from epithelial cell glycoconjugates has been shown to facilitate *T. forsythia*’s adhesion to and invasion of epithelial cells. Human salivary glycoproteins are no exception; they contain various complex sugar substrates, such as mucin. Sialic acid is linked to N-acetylglucosamine through a 2-6′ glycosidic linkage [30,31].

Other authors have hypothesized that, like other human pathogenic bacteria, *T. forsythia* may hydrolyze sugar substrates available in the oral cavity to obtain nutrients and energy during dental biofilm formation. Recently, it has been shown that sialic acid is present on the surface of the oral opportunistic pathogen *Fusobacterium nucleatum*. *T. forsythia* aggregates with *F. nucleatum* and forms synergistic mixed biofilms [25]. The relationship between biofilm formation and virulence is well-described and well-established for several important pathobionts, including *Escherichia coli* and *Pseudomonas aeruginosa*, as well as anaerobic and oral pathogens/pathobionts, such as *T. forsythia*. The ability to adapt to life in oral plaque biofilms (interspecies interactions, evasion of host defenses, and acquisition of local nutrients) is closely related to pathogenicity.

*T. forsythia* is a microorganism that depends on an external supply of N-acetylmuramic acid (MurNAc) for growth. MurNAc is an essential sugar found exclusively in the bacterial peptidoglycan layer [32,33]. Ruscito et al. described the entire peptidoglycan synthesis pathway and identified the missing enzymes in *T. forsythia*. *T. forsythia*’s MurNAc auxotrophy is due to the absence of two key enzymes involved in the de novo synthesis of MurNAc from simple sugars: UDP-N-acetylglucosamine enolpyruvyl transferase (MurA) and UDP-N-acetylenolpyruvoylglucosamine reductase (MurB) [34]. The observed auxotrophy was also evident in its genome analysis: *T. forsythia* lacks essential genes for de novo biosynthesis of peptidoglycan.

In the oral cavity, this bacterium overcomes its auxotrophy by living in multispecies bacterial communities. From these communities, it obtains N-acetylmuramic acid (MurNAc) and other peptidoglycan fragments released during cell division and cell wall rupture. When present in a biofilm, the bacterium can use sialic acid instead of NAM for growth. Thus, the sialic acid utilization system plays a critical role in the bacterium’s survival when an exogenous source of NAM is absent. This system may also play a fundamental role in the formation of subgingival biofilms and the infection of mucosal epithelial cells [35,36].

Thus, an extract that provides muramic acid and/or other peptidoglycan residues to supplement an anaerobic culture medium would be an effective strategy for isolating *T. forsythia*.

Furthermore, to study bacterial sensitivity, analyze bacterial growth characteristics in biofilms, and perform growth curves and qPCR, it is essential to have pure cultures in the laboratory. This necessarily depends on an external supply of MurNAc.

This study aimed to isolate pure cultures of *T. forsythia* by adding an exogenous supply of peptidoglycan (N-acetylmuramic acid) from a common Gram-positive bacterium in clinical microbiology laboratories to the standard anaerobic isolation medium (Fastidious Anaerobe Agar, FAA). For this reason, we selected *S. aureus* ATCC 29213, a commonly used standard quality-control strain in laboratory testing, as the primary source of MurNAc.

To standardize the extraction method, the concentrations of the bacterial lysates were verified via spectrophotometric analysis. A comparative analysis was performed by constructing standard curves using a commercial muramic acid solution and an inexpensive laboratory reagent, lactic acid, to avoid using commercial muramic acid. This allowed us to measure the muramic acid concentrations obtained from the lysate using the lactic acid curve.

## 2. Materials and Methods

### 2.1. Strains

Reference strains from the American Type Culture Collection were used. The *Staphylococcus aureus* strain ATCC 29213 was used to produce muramic acid. The *Tannerella forsythia* strain (ATCC 43037) was used for the *Tannerella* culture.

### 2.2. Obtaining Muramic Acid and Derivatives

A 10-mL volume of an overnight culture of *Staphylococcus aureus* in trypticase soy broth (Scharlau Microbiology, Barcelona, Spain), maintained at 37 °C with an optical density (OD) of 2 at a wavelength of 600 nm, corresponding to a concentration of 3 × 10^9^ colony-forming units (CFUs) per milliliter, was used.

The culture was then centrifuged at 3000 g for eight minutes. The resulting precipitate was resuspended in 2 mL of 1/4 Ringer’s solution containing 2.5 mg/mL lysozyme (L7651, Sigma-Aldrich, St. Louis, MO, USA) and incubated at 37 °C for 24 h to cleave the β-1,4-glycosidic peptidoglycan linkages in the Gram-positive cocci cell wall and release free MurNAc.

Then, proteinase K (P6556-100MG, Sigma-Aldrich, St. Louis, MO, USA) was added to the mixture at a final concentration of 5 mg/mL. The mixture was incubated at 56 °C for 24 h to digest protein debris and remove contaminants from the preparations. The lysate was then subjected to acid hydrolysis with 1 M sulfuric acid at a vol:vol ratio and heated at 100 °C for 2 h. Finally, the resulting lysate was sterilized by filtration through 0.22 μm filters (Millipore), and sterility control was performed by seeding 10 µL of the filtrate in trypticase soy agar (TSA) (Scharlau, Microbiology) at 37 °C for 24 h.

### 2.3. Spectrophotometric Determination of N-Acetylmuramic Acid or MurNAc

The following solutions were used: Muramic acid (M2503-5MG, Sigma-Aldrich): 1 mg/mL; lactic acid (131034, Panreac Chemicals, S.A, Barcelona, Spain): 1 mg/mL; concentrated H_2_SO_4_ (231-639-5, Panreac Chemicals, S.A.): 1 M H_2_SO_4_; 4-phenylphenol (A10817.22, Thermo Scientific™, Waltham, MA, USA), liquefied before use, 1.5% (*m*/*v*) in 96% ethanol; and 4% (*m*/*v*) aqueous CuSO_4_·5H_2_O (844, A339787, Merck, Darmstadt, Germany).

Muramic acid solutions (Sigma) of 10–40 µg/mL were prepared in ground glass-stoppered tubes. Then, 0.5 mL of 1 M sulfuric acid was added to the tubes at a temperature of 100–105 °C for two hours.

Solutions of 10–40 µg/mL lactic acid were prepared in ground glass tubes with ground glass stoppers. For the subsequent conversion of lactic acid to acetaldehyde, 5.5 mL of concentrated sulfuric acid was carefully added to all tubes, which were then shaken vigorously. The tubes were stoppered tightly and heated in a water bath at 100–105 °C for 30 min.

To develop the colorimetric reaction, the tubes were cooled under running water for a few minutes. Then, 50 µL of a 4% copper sulfate solution and 100 µL of a 1.5% 4-phenylphenol solution were added to the tubes, which were then capped, shaken, and incubated at 30 °C for 30 min. After 30 min, the tubes were cooled and the absorbance was measured at 560 nm. A blank solution containing concentrated H_2_SO_4_, 4% CuSO_4_, and 1.5% 4-phenylphenol was used in a spectrophotometer (Peak Instruments Model C-700 UV; Biogen Scientific, S.L, Madrid, Spain). The obtained lysates were analyzed via spectrophotometry and characterized via MALDI-TOF (MALDI Biotyper; Bruker Daltonics, Spain) and mass spectrometry (LTQ Orbitrap Velos, Thermo Fisher Scientific, Waltham, MA, USA).

### 2.4. Pure Culture of T. forsythia

Fastidious Anaerobic Agar (FAA) plates (LabM, Sigma-Aldrich, St. Louis, MO, USA) were coated with 0.1 mL of the obtained lysate containing 12 µg/mL muramic acid. This was done on the basis that *T. forsythia* growth is stimulated by 10 µg/mL N-acetylmuramic acid (MurNAc), as well as by decreasing concentrations of 10, 5, 2.5, and 1 µg/mL muramic acid. The plates were then allowed to dry for 30 min. These MurNAc-containing plates were named FAA Mur agar. Simultaneously, plates were prepared with commercial muramic acid (Sigma) at the same concentrations as the control.

The *T. forsythia* strain ATCC 43037 was cultured in Fastidious Anaerobic Broth (FAB) (LabM, London, UK), supplemented with 10 µg/mL MurNAc, and incubated at 37 °C under anaerobic conditions in a Whitley DG250 anaerobic chamber for 7 days. Finally, 100 μL aliquots of the grown culture were used to inoculate the FAA Mur.

## 3. Results

### 3.1. Obtaining Cell Wall Derivatives

After obtaining cell wall derivatives by enzymatic and acid lysis, approximately 3.5 mL of filtered extract was obtained. An aliquot of this extract was inoculated to ensure that there was no contamination with the original *S. aureus* strain. All of the obtained extracts were free of contaminants.

Next, the amount of muramic acid in the extract was analyzed *via* spectrophotometric determination, as described below. These results allow us to determine how much extract must be added to the anaerobic culture media to isolate *T. forsythia* in pure culture.

### 3.2. Spectrophotometric Determination of N-Acetylmuramic Acid (MurNAc)

Standard curves constructed using a solution of N-acetylmuramic acid in the range of 10–40 µg showed direct proportionality between absorbance and the analyzed values, with the equation y = 0.0099x − 0.045 (R^2^ = 0.9973).

**Figure 1:**
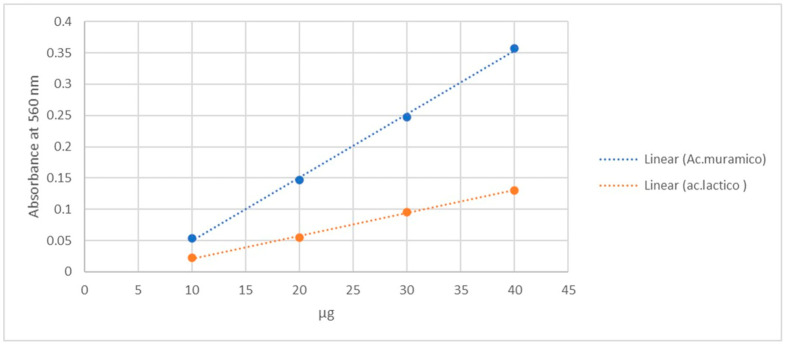
Calibration curves obtained in parallel of lactic acid and muramic acid in the range of 10–40 µg.

The calibration curve obtained using lactic acid, which is feasible for use in any microbiology laboratory, also showed proportionality. The equation of this graph is y = 0.0037x − 0.0155, with R^2^ = 0.9983, as shown in Figure 1.

Muramic acid quantification in the lysate was performed *via* spectrophotometric analysis using the modified Barker and Summerson method. This method is based on the reaction of the lactic acid fraction of the MurNAc molecule, followed by the detection of acetaldehyde released by hydrolysis *via* colorimetry [37]. The results revealed an optical density (OD) of 0.073, which corresponded to 12 µg/mL of muramic acid.

Additionally, a linear regression model based on the relationship between micrograms of lactic acid and its wavelength at 560 nm was used to estimate the wavelength of muramic acid. The following estimation model was obtained:

y = 0.007371 μg + 0.695652ODL − 0.034217

This model correctly predicted the amount of muramic acid present, with a mean absolute error (MAE) of 0.56% and an R^2^ value of 0.997418.

**Figure 2:**
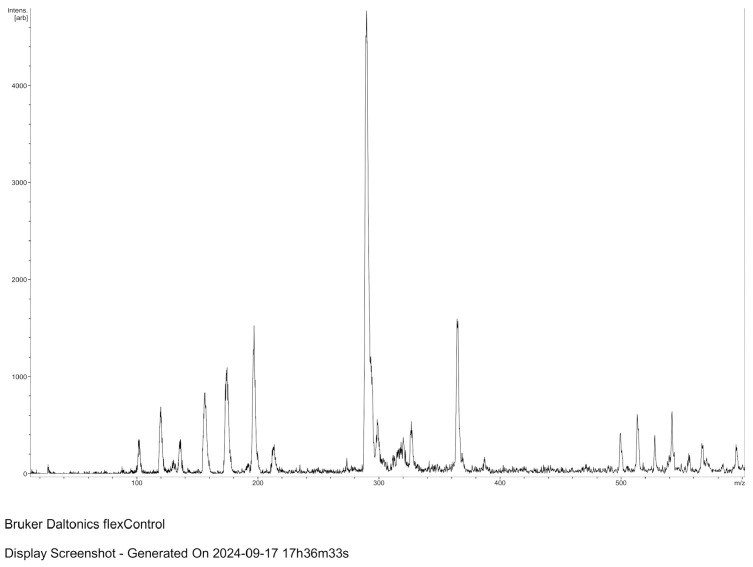
MALDI–TOF spectroscopic analysis of the lysate material (MALDI Biotyper; Bruker Daltonics, Spain).

**Figure 3:**
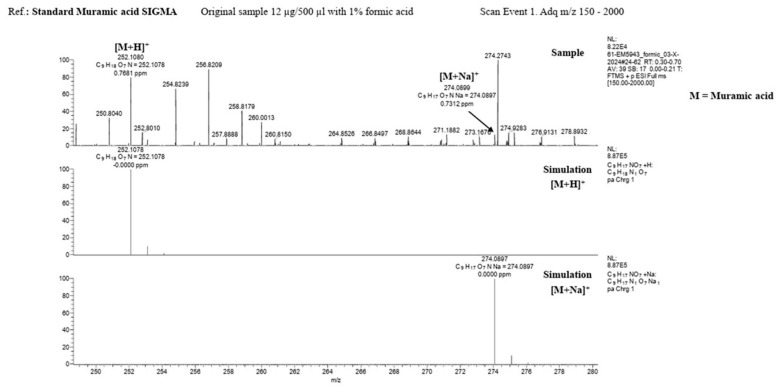

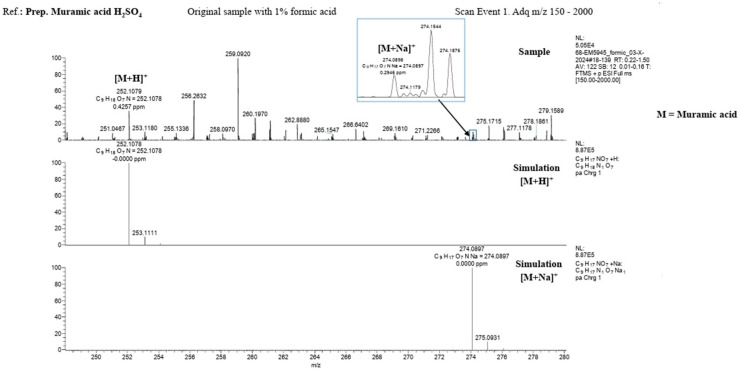
Mass spectrometry (LTQ Orbitrap Velos, Thermo Fisher Scientific) **Upper**: Standard Muramic acid from Sigma and **Down**: lysate material.

MALDI-TOF spectroscopic analysis and mass spectrometry of the lysate material, as well as of commercial muramic acid, showed consistent results, with one peak indicating the presence of muramic acid. Figure 2 and Figure 3.

### 3.3. Pure Culture of T. forsythia

**Figure 4:**
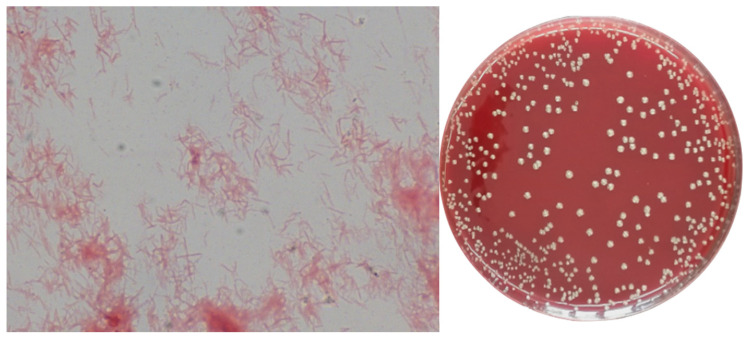
(FAA-Mur) Gram stain and pure culture of *T. forsythia* (The image was taken at 1000×).

Passages were transferred from a liquid medium (FAB-Mur) to a solid medium (FAA-Mur), in which the collection strain of *T. forsythia* (ATCC 43037) was maintained. After incubating for 48 h under anaerobic conditions, tiny, translucent white colonies with convex morphology (sometimes with a central umbilication or “donut” appearance) grew. These colonies corresponded to Gram-negative bacilli on Gra m stain Figure 4.

After obtaining the isolates in the new solid medium FAA Mur, the colonies were identified to confirm that they were *T. forsythia*. The Rapid ID Ana II system was used for this identification. Additionally, qPCR with *T. forsythia* specific primers was performed to assess the identification. See the protocol in Appendix A. Furthermore, it was demonstrated that the strain retained viability through successive passages in Fastidious Anaerobic Broth (FAB) and FAA, both supplemented with a 12 µg/mL solution of MurNAc derived from the lysate of *S. aureus* ATCC 29213.

We did not find any differences when we compared the effects of using 12 µg/mL of commercial (Sigma) MurAc and the obtained lysates by performing colony-forming unit (CFU) counts in parallel. In both cases, the number of *T. forsythia* colonies was approximately 10^8^ (CFU/mL). *T. forsythia* continued to grow when different concentrations of muramic acid were used in the supplement, up to a concentration of 2.5 µg/mL. No colonies were observed at a concentration of 1 µg/mL (three replicates were performed).

## 4. Discussion

This study aimed to present a simple, inexpensive method for routinely isolating and handling *T. forsythia* in the laboratory. Our previous observation that *T. forsythia* grows optimally in a liquid culture medium with a coagulase-negative staphylococcal strain, even without muramic acid supplementation, informed this study.

However, muramic acid supplementation is essential in the study of *T. forsythia*. Allowing this microorganism to grow independently could facilitate its isolation and establish its involvement in pathological processes. Currently, its detection is based on molecular methods, such as polymerase chain reaction (PCR) and ELISA [38]. Bacterial isolation is necessary for standardizing calibration curves. Moreover, isolating a pure culture of *T. forsythia* allows one to perform antimicrobial susceptibility testing.

Some authors describe methods for obtaining muramic acid from Gram-positive or Gram-negative bacteria [39]. These methods use toxic and difficult-to-handle reagents, such as 6 N HCl and phenol. Additionally, the obtained muramic acid was quantified using hydrophilic interactions coupled with gas chromatography–mass spectrometry. This method is very complex and beyond the capabilities of most laboratories.

Conversely, authors such as Mayer et al. [40] emphasize the importance of supplementing *T. forsythia* culture media for various studies. Specifically, these authors used various sources of muramic acid, including *P. gingivalis* and *F. nucleatum* culture supernatants as both species naturally release MurNAc-containing fragments able to sustain *T. forsythia* growth.

The bacterial community that forms the oral biofilm undergoes many metabolic and quorum-sensing exchanges, which means that many oral species that appear to be unculturable actually have special requirements that are met by community growth but not by individual in vitro cultures. *T. forsythia* exhibits satellites when growing around a *Cutibacterium acnes* streak. In fact, the current German DSMZ culture collection provides strain DSM 102835 on a Medium 1203 plate in coculture with *C. acnes* DSM 1897 [41]. *T. forsythia* also exhibits satellites around *P. gingivalis* and *F. nucleatum* in periodontal samples from patients. However, the colonies appear larger when grown with Gram-positive bacteria.

We prepared homemade peptidoglycan-containing solutions and used them as a source of MurNac in the liquid medium culture of an ATCC 29213 strain of *S. aureus*. We then calibrated the measurements using curves with lactic acid instead of muramic acid, as lactic acid is a common reagent in laboratories.

Our findings may be related to *T. forsythia*’s strategy to meet its MurNAc requirements in the oral cavity by utilizing compounds released by coexisting bacteria. Hottman et al. [42] reported that certain bacteria with which *T. forsythia* coexists in the oral cavity, such as *P. gingivalis* and *F. nucleatum*, lack an AmpG orthologue. This prevents them from recovering disaccharides, such as GlcNAc-MurNAc and GlcNAc-anhydroMurNAc, that are released during cell wall turnover. Therefore, these bacteria likely provide *T. forsythia* with essential peptidoglycan fragments.

It has also been suggested that *T. forsythia* may use intact peptidoglycan as a growth factor [43]. An in vitro study of multispecies oral biofilms revealed that *P. gingivalis* OMZ925 strongly colocalized with *T. forsythia* ATCC 43037 [44]. Similarly, *T. forsythia* has been shown to co-aggregate and form synergistic co-biofilms with *F. nucleatum* in vitro and in human dental plaque [45,46]. These observations may be due to a mechanism that ensures the survival of *T. forsythia*. Another possible explanation is that the bacterium depends on neighboring species for additional metabolites and growth-supporting factors released within multispecies biofilms, which further enhances its survival.

Several authors have previously suggested that *T. forsythia* could use other bacteria as a source of muramic acid. In this study, however, we considered a simple way to prepare indigenous peptidoglycan solutions via the liquid culture of a well-known bacterium commonly found in microbiology laboratories. We used the *S. aureus* strain ATCC 29213 as a source of N-acetylmuramic acid (MurNac). We chose *S. aureus* as our source because of its well-documented ability to synthesize this compound, an integral part of its cell wall [47,48]. *S. aureus* has a high proportion of peptidoglycan in its cell wall, suggesting that muramic acid production in these strains is significantly greater than in Gram-negative bacteria [49,50].

We chose to use calibration curves based on lactic acid concentration rather than muramic acid concentration to quantify the amount of muramic acid obtained because lactic acid is a commonly used reagent in microbiology laboratories. The analysis was performed according to the modified Barker and Summerson method, which is based on the reaction of the lactic acid moiety of the MurNAc molecule and the subsequent colorimetric detection of acetaldehyde released by hydrolysis [37,51]. Furthermore, using a linear regression model based on the relationship between micrograms of lactic acid and its wavelength, we can correctly predict the amount of muramic acid in the lysate with a mean absolute error of 0.56% and an R^2^ value of 0.997418. This analytical approach strengthens the accuracy of our measurements and provides a solid basis for interpreting the results of our study. These regression parameters correspond to the calibration curves shown in Figure 1, where both MurNAc and lactic acid standard curves are presented.

Using lactic acid as an alternative to measure and standardize muramic acid solutions conserves laboratory resources while ensuring the quality and reproducibility of experimental preparations. Lactic acid was chosen not only due to its availability but because its chemical structure reproduces the behavior of the lactic acid moiety of MurNAc in the colorimetric assay, making it a reliable and inexpensive surrogate standard. This innovative approach can significantly impact the study of *T. forsythia* by simplifying its cultivation and reducing the associated costs.

While *T. forsythia* growth is stimulated by 10 µg/mL N-acetylmuramic acid (MurNAc), our experiments revealed that as little as 2.5 µg/mL MurNAc is sufficient to promote auxotrophic bacterial growth.

Our results suggest that axenic culture using microbially derived muramic acid is straightforward and enables antimicrobial susceptibility testing, biofilm establishment for evaluating new oral care products, and growth curves for qPCR analysis. These results could greatly help future research into the behavior and biology of *T. forsythia*.

## 5. Conclusions

This study demonstrates that axenic cultures of *Tannerella forsythia* can be reliably obtained by supplementing conventional anaerobic media with a cellular lysate derived from *Staphylococcus aureus*. The method provides a practical and low-cost source of N-acetylmuramic acid (MurNAc), and the lysate can be quantified using calibration curves based on lactic acid concentration, taking advantage of the colorimetric reaction of the lactic acid moiety of the MurNAc molecule.

This strategy simplifies routine laboratory work with *T. forsythia* and enables broader access to pure cultures, which are essential for antimicrobial susceptibility testing, biofilm studies, and the development of growth curves for molecular analyses such as qPCR.

Future studies could explore alternative Gram-positive sources of muramic acid and further characterize the specific peptidoglycan fragments involved in supporting *T. forsythia* growth.

## Data Availability

The data presented in this study are available on request from the corresponding author. The data are not publicly available due to ethical and privacy restrictions.

## Appendix A

Real-Time Quantitative PCR (qPCR) for *Tannerella forsythia*

DNA was extracted using the QIAamp DNA Mini Kit (QIAGEN, Valencia, CA), following the manufacturer’s instructions. The concentration and purity of the DNA were determined spectrophotometrically using a NanoDrop One system (Thermo Scientific, Waltham, MA, USA).

*T. forsythia* quantification was carried out using a real-time polymerase chain reaction (PCR) with a TaqMan probe-based system. Reactions were prepared in a final volume of 10 µL using qPCR Master Mix (Sigma-Aldrich, St. Louis, MO, USA) and processed in a Bioer FQD-48A thermocycler (Hangzhou, China).

The primers (Invitrogen, Custom Primers, Thermo Scientific, Waltham, MA, USA) were as follows: Forward (5′–3′): GGGTGAGTAACGCGTATGTAACCT. Reverse (5′–3′): ACCCATCCGCAACCAATAAA. The specific probe (Sigma-Aldrich, St. Louis, MO, USA), labelled with [6FAM] and [TAM], was: [6FAM] CCCGCAACAGAGGGATAACCCGG [TAM]. Amplification conditions included an initial denaturation at 95 °C for 10 min, followed by 40 cycles of denaturation at 95 °C for 15 s and an extension step at 55 °C for 1 min.

This assay confirmed that the bacterium obtained from culture corresponded to *T. forsythia*.

## References

[1] Kwon T.H., Lamster I.B., Levin L. (2021). Current Concepts in the Management of Periodontitis. Int. Dent. J..

[2] Dannewitz B., Holtfreter B., Eickholz P. (2021). Periodontitis—Therapy of a widespread disease. Bundesgesundheitsblatt-Gesundheitsforschung-Gesundheitsschutz.

[3] Benahmed A.G., Gasmi A., Noor S., Semenova Y., Emadali A., Dadar M., Shanaida Y., Avdeev O., Bjørklund G. (2025). Hallmarks of Periodontitis. Curr. Med. Chem..

[4] Veras E.L., dos Santos N.C., Souza J.G.S., Figueiredo L.C., Retamal-Valdes B., Barão V.A.R., Shibli J., Bertolini M., Faveri M., Teles F. (2023). Newly identified pathogens in periodontitis: Evidence from an association and an elimination study. J. Oral Microbiol..

[5] Socransky S.S., Haffajee A.D., Cugini M.A., Smith C., Kent R.L. (1998). Microbial complexes in subgingival plaque. J. Clin. Periodontol..

[6] Santi-Rocca J., Martín-García D.F., Lorca-Alonso I., la Fuente S.G.-D., Aguado B., Bonner M., Amard V., Amiot P., Bar C., Berbon V. (2025). Microbial Complexes in Subgingival Plaque: A Bacterial Meta-Taxonomic Study. J. Clin. Periodontol..

[7] Wang T., Ishikawa T., Sasaki M., Chiba T. (2022). Oral and Gut Microbial Dysbiosis and Nonalcoholic Fatty Liver Disease: The Central Role of *Porphyromonas gingivalis*. Front. Med..

[8] Li C., Yu R., Ding Y. (2022). Association between *Porphyromonas gingivalis* and systemic diseases: Focus on T cells-mediated adaptive immunity. Front. Cell. Infect. Microbiol..

[9] Chopra A., Bhat S.G., Sivaraman K. (2020). *Porphyromonas gingivalis* adopts intricate and unique molecular mechanisms to survive and persist within the host: A critical update. J. Oral Microbiol..

[10] Zhang J., Xie M., Huang X., Chen G., Yin Y., Lu X., Feng G., Yu R., Chen L. (2021). The Effects of *Porphyromonas gingivalis* on Atherosclerosis-Related Cells. Front. Immunol..

[11] Lai Y., Chu L. (2008). Novel mechanism for conditional aerobic growth of the anaerobic bacterium *Treponema denticola*. Appl. Environ. Microbiol..

[12] Ruscitto A., Hottmann I., Stafford G.P., Schäffer C., Mayer C., Sharma A. (2016). Identification of a novel N-acetylmuramic acid transporter in *Tannerella forsythia*. J. Bacteriol..

[13] Tanner A.C.R., Izard J. (2006). *Tannerella forsythia*, a periodontal pathogen entering the genomic era. Periodontol. 2000.

[14] Tanner A.C.R., Haffer C., Bratthall G.T., Visconti R.A., Socransky S.S. (1979). A study of the bacteria associated with advancing periodontitis in man. J. Clin. Periodontol..

[15] Tran S.D., Rudney J.D., Sparks B.S., Hodges J.S. (2001). Persistent Presence of *Bacteroides forsythus* as a Risk Factor for Attachment Loss in a Population with Low Prevalence and Severity of Adult Periodontitis. J. Periodontol..

[16] Carrouel F., Viennot S., Santamaria J., Veber P., Bourgeois D. (2016). Quantitative molecular detection of 19 major pathogens in the interdental biofilm of periodontally healthy young adults. Front. Microbiol..

[17] Jervøe-Storm P.M., Jepsen S., Jöhren P., Mericske-Stern R., Enkling N. (2015). Internal bacterial colonization of implants: Association with peri-implant bone loss. Clin. Oral Implant. Res..

[18] Tiwari S., Saxena S., Kumari A., Chatterjee S., Hazra A., Choudhary A. (2020). Detection of Red complex bacteria, *P. gingivalis*, *T. denticola* and *T. forsythia* in infected root canals and their association with clinical signs and symptoms. J. Fam. Med. Prim. Care.

[19] Gomes B.P.F.A., Jacinto R.C., Pinheiro E.T., Sousa E.L.R., Zaia A.A., Ferraz C.C.R., Souza-Filho F.J. (2006). Molecular Analysis of *Filifactor alocis, Tannerella forsythia*, and *Treponema denticola* Associated With Primary Endodontic Infections and Failed Endodontic Treatment. J. Endod..

[20] Gomes B.P.F.A., Montagner F., Jacinto R.C., Zaia A.A., Ferraz C.C.R., Souza-Filho F.J. (2007). Polymerase Chain Reaction of *Porphyromonas gingivalis*, *Treponema denticola*, and *Tannerella forsythia* in Primary Endodontic Infections. J. Endod..

[21] Schäffer C., Andrukhov O. (2024). The intriguing strategies of *Tannerella forsythia’s* host interaction. Front. Oral Health.

[22] Bloch S., Hager-Mair F.F., Bacher J., Tomek M.B., Janesch B., Andrukhov O., Schäffer C. (2024). Investigating the role of a *Tannerella forsythia* HtrA protease in host protein degradation and inflammatory response. Front. Oral Health.

[23] Książek M., Goulas T., Mizgalska D., Rodríguez-Banqueri A., Eckhard U., Veillard F., Waligórska I., Benedyk-Machaczka M., Sochaj-Gregorczyk A.M., Madej M. (2022). A unique network of attack, defence and competence on the outer membrane of the periodontitis pathogen *Tannerella forsythia*. Chem. Sci..

[24] Hasebe A., Yoshimura A., Into T., Kataoka H., Tanaka S., Arakawa S., Ishikura H., Golenbock D.T., Sugaya T., Tsuchida N. (2004). Biological Activities of *Bacteroides forsythus* Lipoproteins and Their Possible Pathological Roles in Periodontal Disease. Infect. Immun..

[25] Honma K., Ruscitto A., Frey A.M., Stafford G.P., Sharma A. (2016). Sialic acid transporter NanT participates in *Tannerella forsythia* biofilm formation and survival on epithelial cells. Microb. Pathog..

[26] Corfield T. (1992). Bacterial sialidases-roles in pathogenicity and nutrition. Glycobiology.

[27] Vimr E.R., Kalivoda K.A., Deszo E.L., Steenbergen S.M. (2004). Diversity of Microbial Sialic Acid Metabolism. Microbiol. Mol. Biol. Rev..

[28] Roy S., Douglas C.W.I., Stafford G.P. (2010). A novel sialic acid utilization and uptake system in the periodontal pathogen *Tannerella forsythia*. J. Bacteriol..

[29] Stafford G., Roy S., Honma K., Sharma A. (2012). Sialic acid, periodontal pathogens and *Tannerella forsythia*: Stick around and enjoy the feast!. Mol. Oral Microbiol..

[30] Mizan S., Henk A., Stallings A., Maier M., Lee M.D. (2000). Cloning and characterization of sialidases with 2-6′ and 2-3′ sialyl lactose specificity from *Pasteurella multocida*. J. Bacteriol..

[31] Settem R.P., Sharma A. (2025). Oral bacterium contributes to periodontal inflammation by forming advanced glycation end products. Infect. Immun..

[32] Wyss C. (1989). Dependence of proliferation of *Bacteroides forsythus* on exogenous N-acetylmuramic acid. Infect. Immun..

[33] Vacariu C.M., Tanner M.E. (2022). Recent Advances in the Synthesis and Biological Applications of Peptidoglycan Fragments. Chem.-A Eur. J..

[34] Ruscitto A., Sharma A. (2018). Peptidoglycan synthesis in *Tannerella forsythia*: Scavenging is the modus operandi. Mol. Oral Microbiol..

[35] Hottmann I., Mayer V.M.T., Tomek M.B., Friedrich V., Calvert M.B., Titz A., Schäffer C., Mayer C. (2018). N-acetylmuramic acid (MurNAc) auxotrophy of the oral pathogen *Tannerella forsythia*: Characterization of a MurNAc kinase and analysis of its role in cell wall metabolism. Front. Microbiol..

[36] Wodzanowski K.A., Hyland S.N., Chinthamani S., Sandles L.M.D., Honma K., Sharma A., Grimes C.L. (2022). Investigating Peptidoglycan Recycling Pathways in *Tannerella forsythia* with N-Acetylmuramic Acid Bioorthogonal Probes. ACS Infect. Dis..

[37] Sesartić L., Hadžija O. (1991). Spectrophotometric determination of N-acetylmuramic acid in complex molecules. Anal. Chim. Acta.

[38] Lara E.S.M., Moyeda A.L.G., Ibarra K.I.J., Moreno N.P.F., Depraect N.E.Z., Flores P.F., Delgado K.N.R., Soto J.M.S. (2023). *Tannerella forsythia*: A periodontopathic pathogen review. Int. J. Appl. Dent. Sci..

[39] Pismennõi D., Kattel A., Belouah I., Nahku R., Vilu R., Kobrin E.G. (2023). The Quantitative Measurement of Peptidoglycan Components Obtained from Acidic Hydrolysis in Gram-Positive and Gram-Negative Bacteria via Hydrophilic Interaction Liquid Chromatography Coupled with Mass Spectrometry. Microorganisms.

[40] Mayer V.M.T., Tomek M.B., Figl R., Borisova M., Hottmann I., Blaukopf M., Altmann F., Mayer C., Schäffer C. (2020). Utilization of different MurNAc sources by the oral pathogen *Tannerella forsythia* and role of the inner membrane transporter AmpG. BMC Microbiol..

[41] Leibniz Institute DSMZ–German Collection of Microorganisms and Cell Cultures (2024). Catalogue entry: *Tannerella forsythia* DSM 102835. Supplied on Medium 1203 in Coculture with Cutibacterium Acnes DSM 1897.

[42] Hottmann I., Borisova M., Schäffer C., Mayer C. (2021). Peptidoglycan Salvage Enables the Periodontal Pathogen *Tannerella forsythia* to Survive within the Oral Microbial Community. Microb. Physiol..

[43] Mayer V.M.T., Hottmann I., Figl R., Altmann F., Mayer C., Schäffer C. (2019). Peptidoglycan-type analysis of the N-acetylmuramic acid auxotrophic oral pathogen *Tannerella forsythia* and reclassification of the peptidoglycan-type of *Porphyromonas gingivalis*. BMC Microbiol..

[44] Bloch S., Thurnheer T., Murakami Y., Belibasakis G.N., Schäffer C. (2017). Behavior of two *Tannerella forsythia* strains and their cell surface mutants in multispecies oral biofilms. Mol. Oral Microbiol..

[45] Sharma A. (2020). Persistence of *Tannerella forsythia* and *Fusobacterium nucleatum* in Dental Plaque: A Strategic Alliance. Curr. Oral Health Rep..

[46] Zijnge V., Van Leeuwen M.B.M., Degener J.E., Abbas F., Thurnheer T., Gmür R., Harmsen H.J.M. (2010). Oral biofilm architecture on natural teeth. PLoS ONE.

[47] Strominger J.L., Park J.T., Thompson R.E. (1959). Composition of the cell wall of *Staphylococcus aureus*: Its relation to the mechanism of action of penicillin. J. Biol. Chem..

[48] Demeester K.E., Liang H., Jensen M.R., Jones Z.S., D’Ambrosio E.A., Scinto S.L., Zhou J., Grimes C.L. (2018). Synthesis of Functionalized N-Acetyl Muramic Acids to Probe Bacterial Cell Wall Recycling and Biosynthesis. J. Am. Chem. Soc..

[49] Reith J., Mayer C. (2011). Peptidoglycan turnover and recycling in Gram-Positive bacteria. Appl. Microbiol. Biotechnol..

[50] Borisova M., Gaupp R., Duckworth A., Schneider A., Dalügge D., Mühleck M., Deubel D., Unsleber S., Yu W., Muth G. (2016). Peptidoglycan recycling in gram-positive bacteria is crucial for survival in stationary phase. mBio.

[51] Hadžija O. (1974). A simple method for the quantitative determination of muramic acid. Anal. Biochem..

